# Abdominal aortic aneurysm repair in Sweden vs. Finland: benefits of population-wide screening

**DOI:** 10.1093/eurheartj/ehae665

**Published:** 2024-10-07

**Authors:** Matti T Laine, Kevin Mani, Kim Gunnarsson, Anders Wanhainen, Reijo Sund, Maarit Venermo

**Affiliations:** Department of Vascular Surgery, University of Helsinki and Helsinki University Hospital, Haartmaninkatu 2, FI-00290 Helsinki, Finland; Department of Surgical Sciences, Uppsala University, Uppsala, Sweden; Department of Surgical Sciences, Uppsala University, Uppsala, Sweden; Department of Surgical Sciences, Uppsala University, Uppsala, Sweden; Institute of Clinical Medicine, Surgery, Kuopio Musculoskeletal Research Unit, University of Eastern Finland, Kuopio, Finland; Department of Vascular Surgery, University of Helsinki and Helsinki University Hospital, Haartmaninkatu 2, FI-00290 Helsinki, Finland

**Keywords:** Abdominal aortic aneurysm, Population screening, Epidemiology, Register study, Cardiovascular mortality, Vascular surgery

## Abstract

**Background and Aims:**

In 2006, screening of 65-year-old men for abdominal aortic aneurysm (AAA) was started in Sweden. Decline in aneurysm-related mortality has been reported since, but aneurysm incidence has been diminishing globally. Neighbouring Finland with similar population structure and health care system has no AAA screening programme. The aim of this study was to compare incidence and results of AAA repair in Sweden and Finland to differentiate the effect of screening from other changes in the epidemiology and treatment of AAA.

**Methods:**

All repairs for intact AAA (iAAA) or ruptured AAA (rAAA) from 1998 to 2017 were identified from national registers, and mortality data for these patients were collected.

**Results:**

A total of 15 927 operations for iAAA were performed in Sweden and 6933 in Finland. In Sweden, the yearly operation volume increased after introduction of screening. Both countries showed a decrease in number of rAAA operations, but the decrease was more pronounced in Sweden. Sweden had a higher proportion of all AAA repairs because of rupture in the start of the study but by the end, the proportions were similar in both countries. Long-term survival improved for 65–79-old men in Sweden after start of screening.

**Conclusions:**

This study reveals improvements in results of AAA repair in Sweden. A decrease in rAAA repair and increase in iAAA repair were evident after AAA screening was started in 2006 and resulted in better outcomes. These changes are likely the result of AAA screening as they cannot be seen in neighbouring Finland that is lacking an AAA screening programme.

## Structured Graphical Abstract

**Structured Graphical Abstract:**
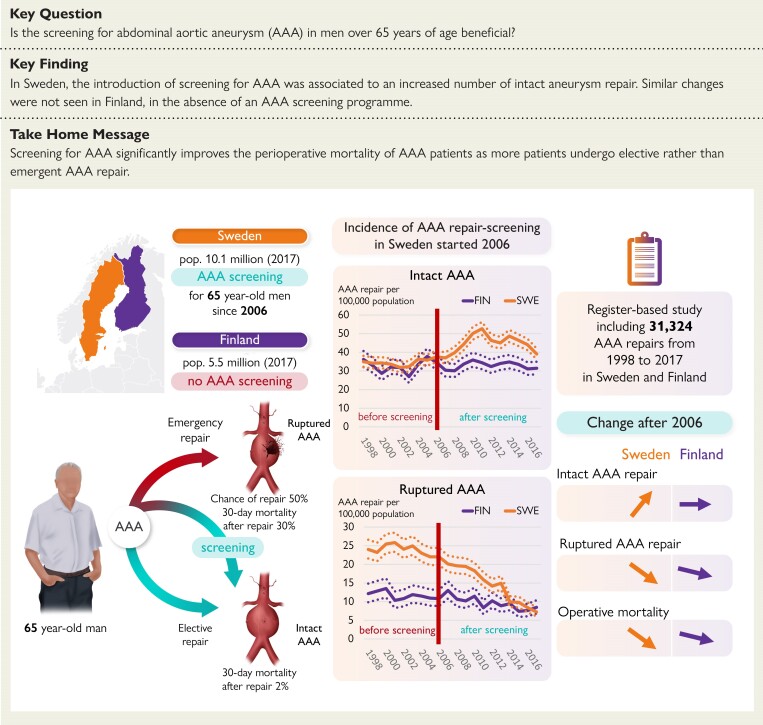
In Sweden, ultrasound screening for AAA in 65-year-old men was started in 2006. This resulted in a rise in number of iAAA repair and a fall in number of emergency repair for rAAA. These changes were not seen in Finland where there is no screening programme for AAA. AAA, abdominal aortic aneurysm; iAAA, intact AAA; rAAA, ruptured AAA.

**See the editorial comment for this article ‘What are the pros and cons of a nationwide abdominal aortic aneurysm screening programme?’, by R. Gottardi *et al*., https://doi.org/10.1093/eurheartj/ehae690.**

## Introduction

Abdominal aortic aneurysm (AAA) is a significant cause of mortality in elderly population.^1,2^ Based on large studies conducted in the 1990s, screening of 65-year-old men was seen as effective for reducing AAA-related mortality by preventing ruptured AAA (rAAA).^3–6^ Screening programmes were therefore initiated in the UK, the USA as part of Medicare, and in Sweden. The Swedish screening programme was introduced gradually starting in 2006, and the programme reached national coverage in 2015. Since the beginning of screening, a decline in rAAA repair has been reported.^7,8^ In Finland, Sweden’s eastern neighbouring country, no screening programmes for AAA were implemented, but aneurysm-related mortality has fallen as well.^9^ This seems to be part of a wider trend throughout many Western countries where AAA incidence is widely reported to be falling.^10^ Likely causes for the fall of AAA prevalence are decrease in number of smokers and improvement in prevention of cardiovascular disease in general, e.g. increased use of statins and antihypertensives.^11^ Aneurysm repair has also changed since the screening trials with the wide introduction of endovascular aneurysm repair (EVAR). This repair method carries a smaller immediate mortality risk compared to open aneurysm repair (OAR), but questions about long-term durability remain.^12^

Finland and Sweden share a similar primarily public health care system and population structure, and both have high-quality national-level register data available. Swedish population in 1998 was 8.9 million and had increased to 10.1 million in 2017. In 1998, 7.3% of population was men 65 years or older; in 2017, their proportion had increased to 9.2%. Population in Finland was 5.2 million in 1998 with 5.6% being men 65 years or older, and in 2017, the respective figures were 5.5 million and 9.4%. Every permanent resident in Finland or Sweden is assigned a personal identity code that can be used to reliably identify patients and match data from different registries.

All 65-year-old men in Sweden, identified in the population registry, are invited to a one-time ultrasound examination. Each region has its own protocol for inviting patients. The examination is performed by specially trained vascular nurses or sonographers. According to a review by the Swedish National Board of Health and Welfare, 79%–83% of the men invited attended screening.^13^ In 2018–22, the prevalence of AAA over 30 mm in diameter in the screened population was .88%.^14^

We compared data on intact AAA (iAAA) and rAAA repair from both countries during a 20-year period. This period has seen the implementation of screening in Sweden—but not in Finland—and adoption of EVAR as primary method of aneurysm repair in both countries. Our aim was to evaluate the effect of these measures on rates and results of AAA repair. Finland serves as a good comparator to Sweden to differentiate effects of screening from other more general trends.

## Methods

Patient-level data from Sweden and Finland were analysed from 1998 to 2017. All operations for iAAA and rAAA repair were identified from national registries and paired using personal identity codes with mortality data from cause-of-death registries. In Finland, the National Care Register for Health Care (HILMO) from the Finnish Institute of Health and Welfare was used to identify all procedures performed for AAA and the cause-of-death register from Statistics Finland was used to identify dates of death. The HILMO registry includes all inpatient and outpatient contacts, including all performed procedures, irrespective of the care provider, and the cause-of-death registry contains data of all mortalities in Finland. Health care providers are required by law to provide these data to the registers. In Sweden, the National Patient Registry (NPR) was employed for identification of all operations, and the national Cause of Death registry for data on date of death. These registries are administered by the National Board of Health and Welfare (NBHW), and they contain data on all inpatient and outpatient care (NPR) and deaths, with registration of diagnoses and surgical procedures based on standardized coding. Extraction of data was performed using diagnostic codes for rAAA and iAAA, according to the International Classification of Disease version 10 (I71.3, I71.4). Surgical procedures were assessed based on the NBHW Classification of surgical procedures and the Nordic Medico-Statistical Committee (NOMESCO) Classification of surgical procedures.^15^

Study was approved by the Institutional Review Board. Anonymized data from both countries were combined and analysed in accordance with the General Data Protection Regulation (GDPR) of the European Union. Only the first procedure of the study period was included in the analysis. Kaplan–Meier method was used for long-term survival analysis. Follow-up started from the index procedure and ended at death (events) or 31 December 2017 (censored) for all Finnish iAAA and rAAA patients and all Swedish rAAA patients. Swedish patients with iAAA operated between 1998 and 2013 were followed up until end of 2014, and those operated from 2014 onwards were followed up until end of 2017.

Yearly operation rates were calculated by dividing the number of operated patients in the reported age group by the reported population in the same age group for the year in question. Population data were obtained from Statistics Sweden and Statistics Finland.^16,17^ Rates are reported as per 100 000 person-years with 95% confidence intervals (CIs) in parentheses.

Hypothetical model for AAA repair incidence in Finland and Sweden was created by calculating the average number of operations per year before screening in Sweden 1998–2005 and using this as baseline incidence. The yearly change from the baseline was calculated for both countries for the following years, and then corresponding relative changes were applied to the baseline of the other country to create a simplified prediction of the scale of change in repair volumes caused by the screening programme. Number of deaths in the hypothetical model was calculated using the actual 90-day mortality rate for iAAA and rAAA repair each year for the corresponding country.

SPSS 29.0 (IBM, Armonk, NY, USA) and STATA 16.0 (StataCorp LLC, Collage Station, TX, USA) were used for statistical analysis.

## Results

From 1998 to 2017, a total of 15 927 operations for iAAA were performed in Sweden and 6933 in Finland. In Sweden, 83% of patients were men and in Finland 86%. Repairs for rAAA numbered 7267 in Sweden and 2197 in Finland, and 82% of patients were men in Sweden and 88% in Finland. Number of operations and proportion of EVAR are shown in *Table 1* for years before and after start of screening in Sweden.

**Table 1:** Total number of operations for intact abdominal aortic aneurysm and ruptured abdominal aortic aneurysm in Sweden and Finland from 1998 to 2017 divided to period before and after the start of abdominal aortic aneurysm screening in Sweden

			1998–2005	2006–17
Sweden	Men	iAAA	4148	9116
		% EVAR	19.3	56.9
		rAAA	2919	3028
		% EVAR	6.3	29.2
	Women	iAAA	857	1806
		% EVAR	15.3	53.0
		rAAA	543	777
		% EVAR	5.5	28.2
Finland	Men	iAAA	2147	2935
		% EVAR	18.6	46.8
		rAAA	779	1149
		% EVAR	1.3	10.0
	Women	iAAA	297	554
		% EVAR	16.2	46.6
		rAAA	116	153
		% EVAR	.0	17.6

Percentage of repairs performed using EVAR is indicated.

### Incidence of intact abdominal aortic aneurysm and ruptured abdominal aortic aneurysm repairs

**Figure 1:**
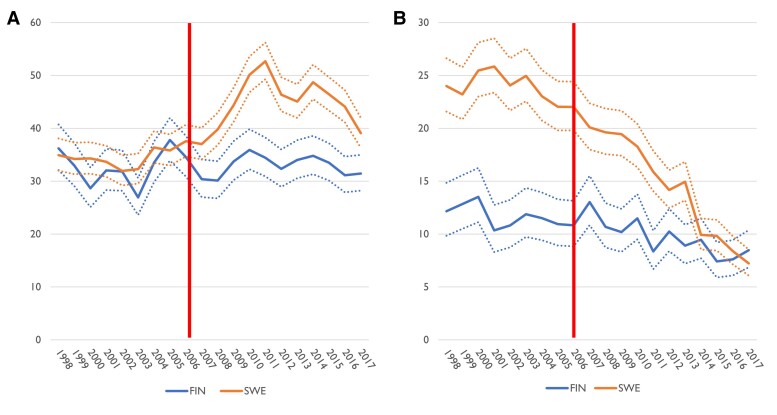
Incidence of abdominal aortic aneurysm repair in Finnish and Swedish men aged 50 years or older per 100 000 person-years. (*A*) Intact abdominal aortic aneurysm repair. (*B*) Ruptured abdominal aortic aneurysm repair. Ninety-five per cent confidence interval is indicated by dashed line. Start of screening in Sweden is indicated by the vertical line. FIN, Finland; SWE, Sweden

**Figure 2:**
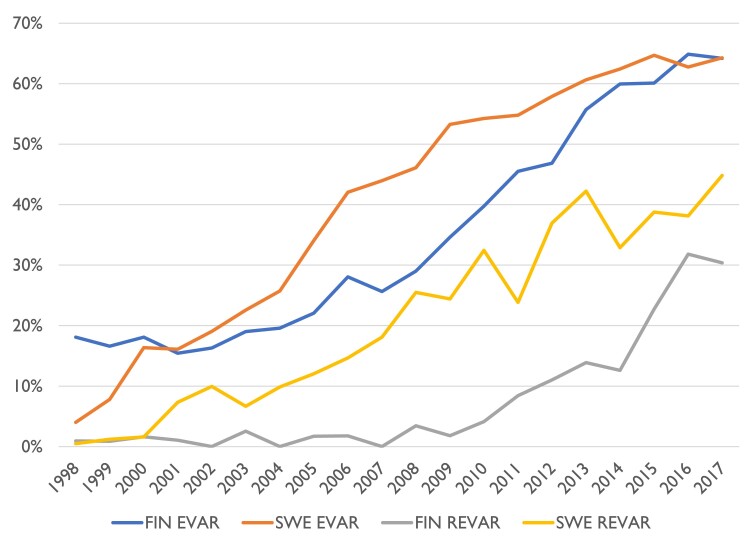
Percentage of abdominal aortic aneurysm repair using endovascular aneurysm repair in Sweden and Finland for intact abdominal aortic aneurysm and ruptured abdominal aortic aneurysm. FIN, Finland; SWE, Sweden; REVAR, EVAR for rAAA

Relative to the population, 40.6 iAAA operations yearly per 100 000 person-years among men over 50 years of age were performed in Sweden (95% CI 39.9–41.3 per 100 000) and 32.8 in Finland (32.0–33.7) during the entire study period. In Sweden, the yearly operation volume increased from 2006 to 2012, i.e. after introduction of screening for AAA in 65-year-old men, while in Finland, the yearly iAAA operation numbers for men stayed stable for the 20-year period (*Figure 1A*). In Swedish men of 65–79 years, the incidence of iAAA repair increased from 72.8 prior to screening (70.2–75.5) to 84.0 after initiation of screening (81.9–86.1). In Finland, there was a small decrease in the number of operations in the same period from 68.1 (64.6–71.8) to 60.1 (57.8–62.5). For men 80 or over, the incidence of iAAA repair increased in both countries, from 40.5 (37.2–44.1) to 71.3 (67.9–74.9) in Sweden and from 54.6 (47.8–62.0) to 82.9 (77.5–88.7) in Finland. The incidence of open iAAA repair in this group decreased slightly from 26.1 (23.5–29.0) to 19.9 (18.1–21.8) in Sweden and from 32.3 (27.2–38.1) to 24.8 (21.9–28.0) in Finland over this period, while EVAR increased from 14.4 (12.4–16.5) to 51.4 (48.5–54.4) in Sweden and 22.3 (18.1–27.1) to 58.1 (53.6–63.0) in Finland. Both countries showed a decrease in number of rAAA operations, but the decrease was more pronounced in Sweden (*Figure 1B*). For men over 50 years, repair rate for rAAA in Sweden prior to screening was 24.1 (23.2–25.0) and after start of screening 14.8 (14.2–15.3); in Finland, rates for corresponding years were 11.7 (10.9–12.6) and 9.7 (9.1–10.2). Adoption of EVAR happened fairly contemporaneously in Sweden and in Finland, but EVAR for rupture repair was adopted earlier in Sweden (*Figure 2*).

### Proportion of women and patients’ age

In 1998–2005, 17.1% (95% CI 16.1%–18.2%) of patients undergoing iAAA repair in Sweden were women and 12.2% (10.9%–13.5%) in Finland. In 2006–17, corresponding percentages were 16.5% (15.4%–17.2%) for Sweden and 12.3% (11.4%–13.3%) for Finland. Of all rAAA repair in Sweden in 1998–2005, 15.7% (14.5%–16.9%) were for women; in Finland, this was 13.0% (10.9%–15.3%). In 2006–17, corresponding percentages were 20.5% (19.2%–21.8%) in Sweden and 11.8% (10.1%–13.6%) in Finland.

**Figure 3:**
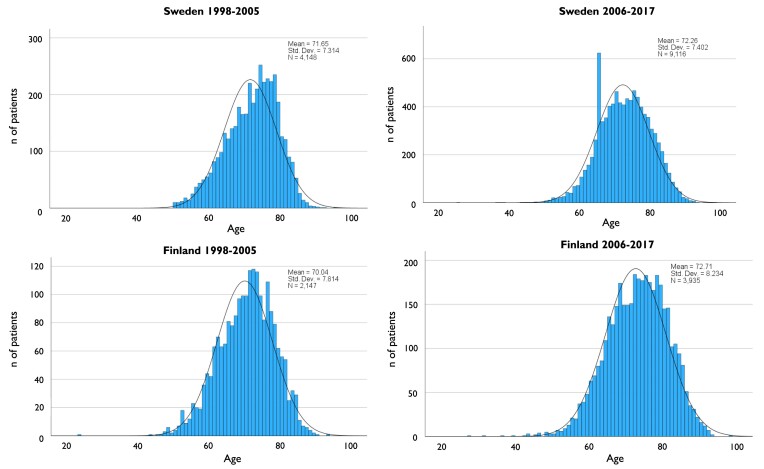
Age distribution of men undergoing intact abdominal aortic aneurysm repair in Sweden and Finland before introduction of screening in Sweden (1998–2005) and after (2006–17). A spike for men of 66 years is seen in Sweden after the introduction of screening

**Figure 4:**
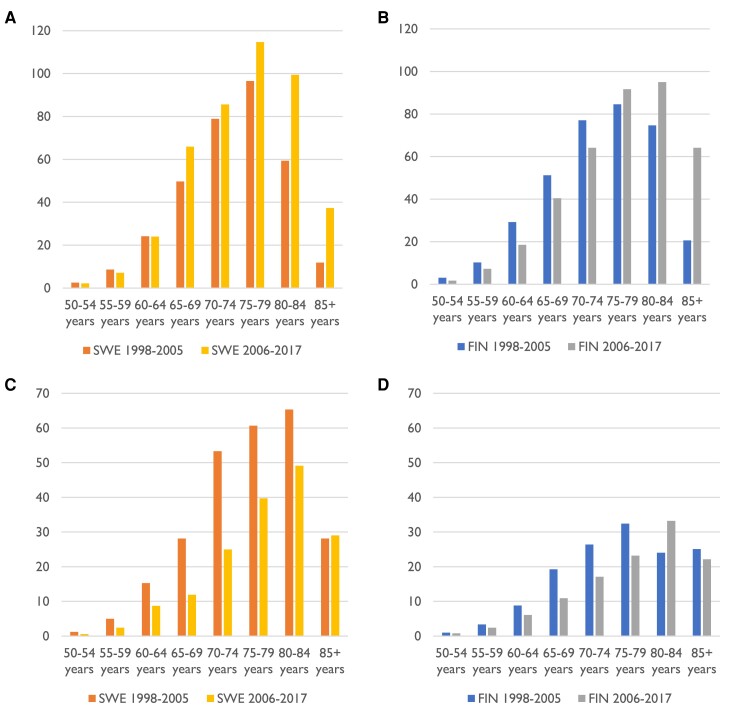
Number of abdominal aortic aneurysm repair per 100 000 person-years in men of different age groups before and after introduction of screening in Sweden. (*A*) Intact abdominal aortic aneurysm repair in Sweden. (*B*) Intact abdominal aortic aneurysm repair in Finland. (*C*) Ruptured abdominal aortic aneurysm repair in Sweden. (*D*) Ruptured abdominal aortic aneurysm repair in Finland. FIN, Finland; SWE, Sweden

For men, there was a statistically significant shift towards older population between 1998–2005 and 2006–17. Men undergoing iAAA repair in 1998–2005 were younger in Finland than in Sweden, but this was reversed for 2006–17. Patients that required repair for rAAA were older than those whose repair was for iAAA, with the exception of the mean age for men in Finland in 2006–17 that was similar for iAAA and rAAA repair. Women were consistently older than men. The mean ages for patients undergoing AAA repair are shown in *Table 2*, and the age distribution for iAAA repair is shown in *Figures 3* and *4*. In the latter part of the study, a clear peak was seen in Swedish men of 65 years as a result of screening programme.

**Table 2:** Mean age (95% confidence interval) for men and women according to country and aneurysm status in the first and last 10-year period of the study

			1998–2005	2006–17
Sweden	iAAA	Men	71.6 (71.4–71.9)	72.3 (72.1–72.4)
		Women	72.9 (72.5–73.4)	73.7 (73.3–74.0)
	rAAA	Men	73.2 (72.9–73.5)	74.5 (74.2–74.8)
		Women	76.3 (75.7–76.9)	76.4 (75.9–76.9)
Finland	iAAA	Men	70.0 (69.7–70.4)	72.7 (72.5–73.0)
		Women	74.7 (73.8–75.6)	76.1 (75.4–76.9)
	rAAA	Men	70.8 (70.2–71.4)	72.9 (72.4–73.4)
		Women	77.6 (76.2–78.9)	78.9 (77.6–80.1)

### Mortality

**Figure 5:**
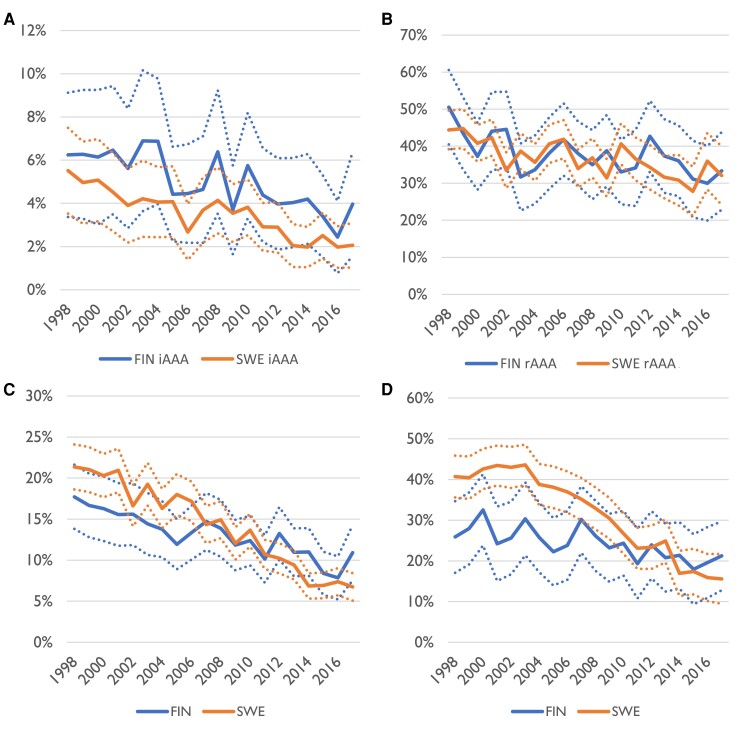
Yearly short-term all-cause mortality for men after abdominal aortic aneurysm repair. (*A*) Ninety-day mortality after intact abdominal aortic aneurysm repair. (*B*) Ninety-day mortality after ruptured abdominal aortic aneurysm repair. (*C*) Ninety-day mortality after all abdominal aortic aneurysm (intact abdominal aortic aneurysm and ruptured abdominal aortic aneurysm) repair. (*D*) Proportion of ruptured abdominal aortic aneurysm of all abdominal aortic aneurysm repairs. Ninety-five per cent confidence intervals shown with dashed lines. FIN, Finland; SWE, Sweden

Annual 90-day mortality for iAAA repair was slightly lower in Sweden, and both countries showed a similar decrease over the 20-year period. In 1998–2005, 90-day mortality in Sweden was 3.4% (95% CI 2.4%–4.8%) after EVAR and 5.0% (4.3%–5.7%) after OAR and 2.5% (1.3%–4.3%) and 7.2% (6.1%–8.5%) in Finland, respectively. In 2006–17, 90-day mortality in Sweden after EVAR was 2.5% (2.2%–3.0%) and 3.9% (3.3%–4.5%) after OAR, and in Finland, it was 2.8% (95% CI 2.1%–3.6%) and 5.8% (4.9%–6.8%), respectively. Mortality after rAAA repair did not differ between the countries. Sweden had a higher proportion of all AAA repairs because of rupture in the start of the study, but by the end, the proportions were similar in both countries (*Figure 5*).

**Figure 6:**
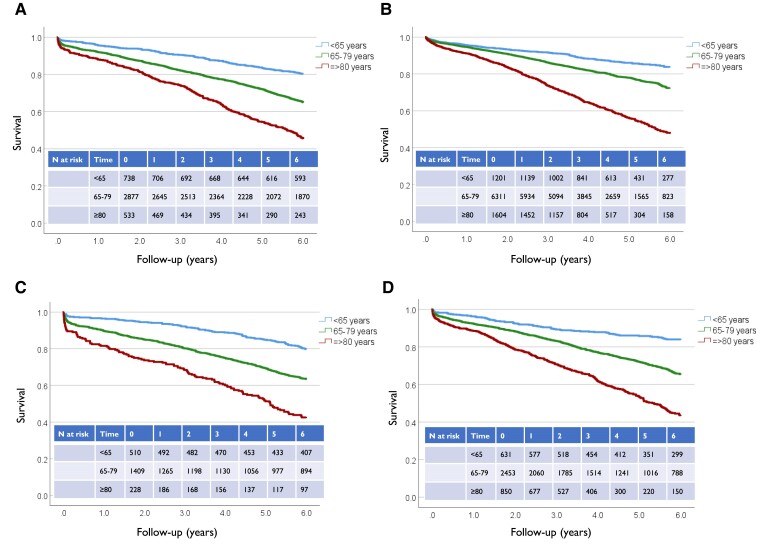
Kaplan–Meier analysis of long-term survival after intact abdominal aortic aneurysm repair for men in three age groups. (*A*) Sweden, year of operation 1998–2005. (*B*) Sweden, year of operation 2006–17. (*C*) Finland, year of operation 1998–2005. (*D*) Finland, year of operation 2006–17. Log rank comparisons and corresponding *P*-values: Sweden 1998–2005 vs. 2006–2017: <65 *P* = .128, 65–79 *P* < .001, ≥80 *P* = .386; Finland 1998–2005 vs. 2006–2017: <65 *P* = .213, 65–79 *P* = .091, ≥80 *P* = .444; 1998–2005 Sweden vs. Finland: <65 *P* = .899, 65–79 *P* = .202, ≥80 *P* = .262; 2006–17 Sweden vs. Finland: <65 *P* = .844, 65–79 *P* < .001, ≥80 *P* = .051

Mortality during 6 years of follow-up was slightly lower in Sweden than in Finland for all men undergoing iAAA repair, but no statistically significant difference could be seen for rAAA. Mean survival after iAAA was 5.0 years (95% CI 5.0–5.1) in Sweden and 4.9 years (4.8–4.9) in Finland. After rAAA repair, mean survival was 3.1 years (3.0–3.2) and 3.2 years (3.0–3.3), respectively. Survival for 65–79-year-old men improved in Sweden after 2006. No statistically significant improvement in this age group was seen in Finland. No difference in survival was evident in men under 65 or over 80 (*Figure 6*).

### Estimation of screening’s effect on perioperative mortality

**Figure 7:**
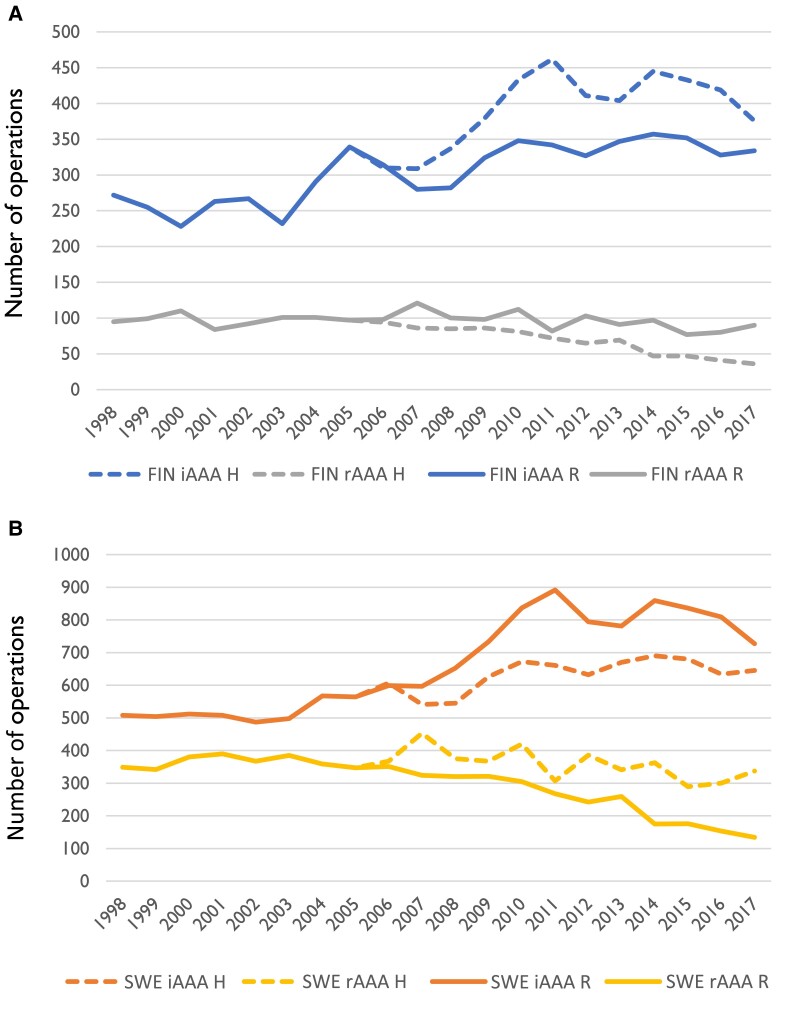
(*A*) Hypothetical model showing similar relative changes in yearly incidence of operations for intact abdominal aortic aneurysm and ruptured abdominal aortic aneurysm in Finland than were seen in Sweden after abdominal aortic aneurysm screening. (*B*) Hypothetical model showing similar relative changes in yearly incidence of operations for intact and ruptured aneurysms in Sweden than were seen in Finland where abdominal aortic aneurysm screening was not implemented. In both scenarios, the mean incidence in 1998–2005 was used as the base level. The actual reported incidence of operations is also shown for comparison. FIN, Finland; SWE, Sweden; H, hypothetical model; R, actual reported incidence

In a hypothetical scenario, if Finland would have launched a national screening programme at the same time as Sweden and if the rate of operations for both iAAA and rAAA would have been affected similarly as in Sweden, there would have been 783 more iAAA repairs and 341 fewer rAAA repairs during 2006–17. This would have translated to 34 additional deaths from iAAA repair and 128 fewer deaths from rAAA repair, thus 94 fewer deaths from AAA repair in total. Conversely, had Sweden not begun screening from 2006 onwards and had the number of operations been similar than in Finland, this would have led to 1514 fewer iAAA repairs and 1277 more rAAA repairs. Deaths from iAAA repair would have been 47 less, but deaths from rAAA repair were 467 more with a net increase of 420 deaths from AAA repair. This crude comparison only considers the mortality from the operations and does not include those patients that would never reach the hospital in the case of rAAA. Yearly hypothetical and real-world number of operations for both countries for rAAA and iAAA are shown in *Figure 7*.

## Discussion

In the current study using national registries, we showed a clear decrease in rAAA repairs in Sweden and, correspondingly, an increase in iAAA repairs. In Finland, the incidence of rAAA repair decreased as well, but less than in Sweden. The total number of aneurysm repairs remained approximately the same, but in Sweden, the proportion of repair done because of rAAA decreased strongly after initiation of the screening programme.

The incidence of iAAA repair was similar in both countries at the start of the study, but increased significantly in Sweden, which can be attributed to screening. The incidence returned closed to the baseline level by the end of the study period. This decrease is likely due to continued declining prevalence of AAA in the population as evidenced by fewer aneurysms being found in the screening programme in more recent years.^11^ Also the initial increase may partly be explained by the possibility of ‘self-referral’ at the start of the programme for those with positive family history for AAA.

The incidence of repair for rAAA was considerably higher in Sweden to begin with and with screening decreased to the same level as in Finland. This can have several possible explanations. This may reflect an actual higher prevalence of AAA in Sweden or be because of other factors. These could include better detection of asymptomatic aneurysms in Finland prior to Swedish screening or higher detection rate and lower pre-hospital mortality or turn-down rate of rAAA patients in Sweden. Smoking is associated not only with incidence of AAA but also with increased rupture risk. Age-standardized tobacco smoking prevalence in men in Finland decreased from 34.3% to 21.2% from 2000 to 2018 and in Sweden from 30.3% to 17.2%.^16,17^ Smoking among men in Finland is more common, although the use of tobacco products in general is higher in Sweden (2000 48.6%, 2018 31.2% vs. 2000 46.7%, 2018 28.3%) due to the use of Swedish type smokeless tobacco.^18^ This, however, has not been shown to be associated with AAA.^19^ Thus, the difference in incidence of rAAA repair between the two countries is unlikely to be explained by different smoking habits, although the decrease in smoking seen in both countries is likely to be one of the factors why rAAA incidence in general seems to be decreasing.

Even though the age of men undergoing iAAA repair in Sweden increased, the increase was smaller compared to Finland (.6 years compared to 2.5 years). This is likely the result of early detection due to screening and would explain the improvement in long-term survival of men 65–79 years of age seen in Sweden but not in Finland and not in other age groups. Proportion of men over 65 in the population increased in both countries during the 20-year period, but more in Finland. Based on VASCUNET data, increasing age of patients treated for iAAA seems to be the trend in the Nordic countries (Sweden, Finland, Norway, and Denmark) and to a lesser extent in Australia but not in Hungary, New Zealand, or the UK.^20,21^ Variations in smoking habits and repair methods may explain some of this. Nordic countries have had a high smoking prevalence in to begin with and a large drop in number of smokers compared to Australia, New Zealand, and the UK, where smoking was not as common and drop in prevalence was smaller. On the other hand, in Hungary, smoking prevalence remains high.

As iAAA repair in Sweden increased in men, a larger proportion of patients presenting with rupture were women. In Finland, the proportion of women undergoing rAAA repair did not change.

In recent years, there has been little enthusiasm for new AAA screening as incidence of AAA is falling.^7,22,23^ There is some interest in more wide-ranging screening including other cardiovascular conditions in addition to AAA such as arterial hypertension, peripheral vascular disease, and coronary artery disease.^24,25^ However, in recent Danish cohort of 65–74-year-old men who underwent cardiovascular screening, no mortality benefit was seen in 5.6-year follow-up.^26^ In our study, a clear difference between Sweden and Finland was seen after initiation of screening in the rAAA repair rate. Comparing the two countries showed that a significant number of lives were saved in Sweden just through changes in perioperative mortality. Thus, according to our study, screening has been beneficial among male population, at least in Sweden. In previous analyses, the Swedish AAA screening programme has also been found to be cost-effective.^27^ Wanhainen *et al.*^7^ estimated that the screening programme would annually result in gain of 577 quality-adjusted life years (QALYs) with incremental cost-efficiency ratio of 7770 euros per QALY.

The strength of the current study is that it provides real-world data on AAA operations capturing the entire population of two neighbouring countries using national registries. Patient-level data could be used for analysis with reliable data on time of death.

Limitations of this study include the questions on the reliability of registry data.^28^ The registries in question are nationwide so the number of missed cases should be minimal. Data in the registry can be mislabelled but given the large number of cases in our study, the effect of this should be negligible when looking at large trends over long time.

## Conclusions

Our results demonstrate that screening for AAA has had an effect in Sweden not only compared to data from before the start of the programme but also to contemporary data from Finland where no screening programmes exist.^7^ The effect has been an increase in iAAA repair and a corresponding fall in rAAA repair. This has positively affected the survival of patients with AAA as significantly higher proportion of repair is performed in lower risk elective setting than as an emergency procedure.

## Supplementary data

Supplementary data are not available at *European Heart Journal* online.

## Data Availability

The data underlying this article cannot be shared publicly due to privacy regulations and requirements of the authorities to store and analyse it on a secure server. The data will be shared on reasonable request to the corresponding author.

## Pre-registered Clinical Trial Number

None supplied.
